# Abundance and Diversity of Dockerin-Containing Proteins in the Fiber-Degrading Rumen Bacterium, *Ruminococcus flavefaciens* FD-1

**DOI:** 10.1371/journal.pone.0012476

**Published:** 2010-08-30

**Authors:** Marco T. Rincon, Bareket Dassa, Harry J. Flint, Anthony J. Travis, Sadanari Jindou, Ilya Borovok, Raphael Lamed, Edward A. Bayer, Bernard Henrissat, Pedro M. Coutinho, Dion A. Antonopoulos, Margret E. Berg Miller, Bryan A. White

**Affiliations:** 1 Microbial Ecology Group, Rowett Institute of Nutrition and Health, University of Aberdeen, Aberdeen, United Kingdom; 2 Department of Biological Chemistry, The Weizmann Institute of Science, Rehovot, Israel; 3 Department of Molecular Microbiology and Biotechnology, Tel Aviv University, Ramat Aviv, Israel; 4 Architecture et Fonction des Macromolecules Biologiques, CNRS and Universites d'Aix-Marseille I & II, Marseille, France; 5 Division of Nutritional Sciences, Department of Animal Sciences, North American Consortium for Genomics of Fibrolytic Ruminal Bacteria, University of Illinois, Urbana, Illinois, United States of America; The J. Craig Venter Institute, United States of America

## Abstract

**Background:**

The cellulosome is a multi-enzyme machine, which plays a key role in the breakdown of plant cell walls in many anaerobic cellulose-degrading microorganisms. *Ruminococcus flavefaciens* FD-1, a major fiber-degrading bacterium present in the gut of herbivores, has the most intricate cellulosomal organization thus far described. Cellulosome complexes are assembled through high-affinity cohesin-dockerin interactions. More than two-hundred dockerin-containing proteins have been identified in the *R. flavefaciens* genome, yet the reason for the expansion of these crucial cellulosomal components is yet unknown.

**Methodology/Principal Findings:**

We have explored the full spectrum of 222 dockerin-containing proteins potentially involved in the assembly of cellulosome-like complexes of *R. flavefaciens*. Bioinformatic analysis of the various dockerin modules showed distinctive conservation patterns within their two Ca^2+^-binding repeats and their flanking regions. Thus, we established the conceptual framework for six major groups of dockerin types, according to their unique sequence features. Within this framework, the modular architecture of the parent proteins, some of which are multi-functional proteins, was evaluated together with their gene expression levels. Specific dockerin types were found to be associated with selected groups of functional components, such as carbohydrate-binding modules, numerous peptidases, and/or carbohydrate-active enzymes. In addition, members of other dockerin groups were linked to structural proteins, e.g., cohesin-containing proteins, belonging to the scaffoldins.

**Conclusions/Significance:**

This report profiles the abundance and sequence diversity of the *R. flavefaciens* FD-1 dockerins, and provides the molecular basis for future understanding of the potential for a wide array of cohesin-dockerin specificities. Conserved differences between dockerins may be reflected in their stability, function or expression within the context of the parent protein, in response to their role in the rumen environment.

## Introduction

Cellulolytic ruminococci play a major role in the breakdown of plant cell wall material in the rumen and in the hindgut of mammals [1]–[5]. Although their cellulolytic enzyme systems have been investigated for many years [6]–[16] the mechanisms by which they achieve plant cell wall breakdown are only now becoming clear. Recent work on two *Ruminococcus flavefaciens* strains, 17 and FD-1 has revealed a cellulosomal type of enzyme complex, in which a number of the known hydrolytic enzymes have been shown to associate with scaffolding proteins ScaA and ScaB, via specific cohesin-dockerin interactions [17]–[20]. The system in *R. flavefaciens*, however, appears more complex than those reported previously in cellulolytic *Clostridium* species [21]–[23] and comprises numerous cohesin-containing scaffoldins (ScaA, ScaB, ScaC and ScaE) together with interacting enzymes and dockerin-containing proteins [24]. The major structural components of the *R. flavefaciens* cellulosome are encoded by the *sca* gene cluster, whose presence has been demonstrated in five different strains of this species [25].

Notably, not all types of dockerins found in enzymes from *R. flavefaciens* interact with ScaA or ScaB, and there are indications that additional cohesin-dockerin specificities and additional scaffolding proteins are involved in assembling these enzymes into complexes [19]. In this context, ScaA was shown to interact with the small adaptor protein, ScaC, which carries a divergent cohesin that recognises a range of thus far unidentified proteins, distinct from those recognised by the ScaA cohesins [26].

The anchoring of the cellulosome complex to the bacterial cell envelope has also been found to differ from the clostridial cellulosome model. A novel single-cohesin scaffolding protein, ScaE, has a C-terminal anchoring domain with a canonical LPXTG motif that is bound covalently to the bacterial peptidoglycan via a sortase-mediated mechanism [27]. The C-terminal dockerin of ScaB interacts specifically with the cohesin of the bacterial cell-wall anchored ScaE, thereby associating the entire complex to the cell surface. Another key feature of the *R. flavefaciens* system is that identifiable carbohydrate-binding modules (CBMs) are absent from the known scaffolding proteins, but the *sca* gene cluster encodes a distinct cellulose binding protein, CttA, which is also bound to ScaE via a C-terminal dockerin [28].

To date, the bulk of our understanding of the *R. flavefaciens* cellulosome system has come from analysis of the *sca* gene cluster. More extensive analysis of the *R. flavefaciens* FD-1 genome [24] indicated that it harbours the largest number of dockerin-containing components known so far, and explored the expression of cellulolytic enzymes via functional microarray analysis. In the present communication, we have analyzed the draft genome sequence of *R. flavefaciens* FD-1, in order to profile the full spectrum of cohesin-dockerin types and the full range of interacting dockerin-containing proteins involved in the assembly of cellulosome-like complexes. In this context, we report herein an analysis of an unprecedented number of dockerin sequences and their flanking regions that have been detected in the *R. flavefaciens* FD-1 draft genome. The sequences of dockerins were thus examined and classified into nine groups with distinct sequence features. In addition, the characteristics of the parent proteins were examined with respect to their modular architecture with a focus on carbohydrate-active modules and gene expression levels. Altogether, the results demonstrate the abundance and variability of the dockerins and suggest the potential for a wide array of cohesin-dockerin specificities.

## Materials and Methods

### Genome sequencing data

*R. flavefaciens* FD-1 genomic DNA was sequenced at the ‘University of Illinois in-house genomic facility’ using a shotgun sequencing approach. Details of the genome assembly and statistics can be seen in Berg Miller *et al* [24].

### Retrieval of dockerin-containing sequences

Contigs from the *R. flavefaciens* FD-1 genome were used to create a local database of nucleotides and translated open reading frames were generated using the heuristic model of GeneMark™-Gene prediction software programs (http://exon.biology.gatech.edu/GeneMark/). A local BLAST search engine (http://www.ncbi.com) was used at the Rowett Research Institute computer facility (openMosix Beowulf cluster) to retrieved dockerin-containing signature sequences [29]. In each case, the search was initiated with a 60-amino-acid sequence of the *R. flavefaciens* strain 17 dockerin from Cel44A (formerly known as EndB, Q934F9), ScaA (Q9AE53), ScaB (Q9AE52), Ce3B (Q9RLB8) and Xyn11E (Q9L3K3).

Sequences were then further analyzed individually to identify obvious modular structures and BLAST searches were carried out on individual modules or entire polypeptides accordingly. Annotation was carried out using the aid of CD-search (http://www.ncbi.nlm.nih.gov/Structure/cdd/wrpsb.cgi), Pfam domain database (http://www.sanger.ac.uk/Software/Pfam/), Carbonydrate active enzyme-CAZY (http://www.cazy.org/) and Interpro database (http://www.ebi.ac.uk/-interpro/). Low-scoring hits were inspected individually by comparing them against known dockerin sequences for alignment of conserved amino acids.

### Categorization of dockerin groups

Dockerins were first compared with each other using BLAST searches, and sequences with significant similarity (E values below 10^−8^) were grouped together. Sequences in each group were aligned (using ClustalW [30] and Dialign [31]) for careful inspection of their features. The similarity of the sequences was manually examined along different segments of the dockerin, including the two Ca^2+^-binding repeats, putative helices and linkers. Logos of the sequences in each group were created with Weblogo v.2.8.2 (http://weblogo.berkeley.edu/). Searches for homologs were done using BLAST against the non-redundant databases.

### Microarray data source

The change in gene expression of *R. flavefaciens FD-1* was determined by Berg Miller *et al* [24] for cultures grown on either cellulose [0.1% w/v pebble milled cellulose (filter paper)] or cellobiose (0.4% w/v) as a carbon and energy substrate. Microarray data was obtained from Table S9 in Berg Miller *et al* [24].

## Results

### Identification of *R. flavefaciens* FD-1 dockerins

**Figure 1:**
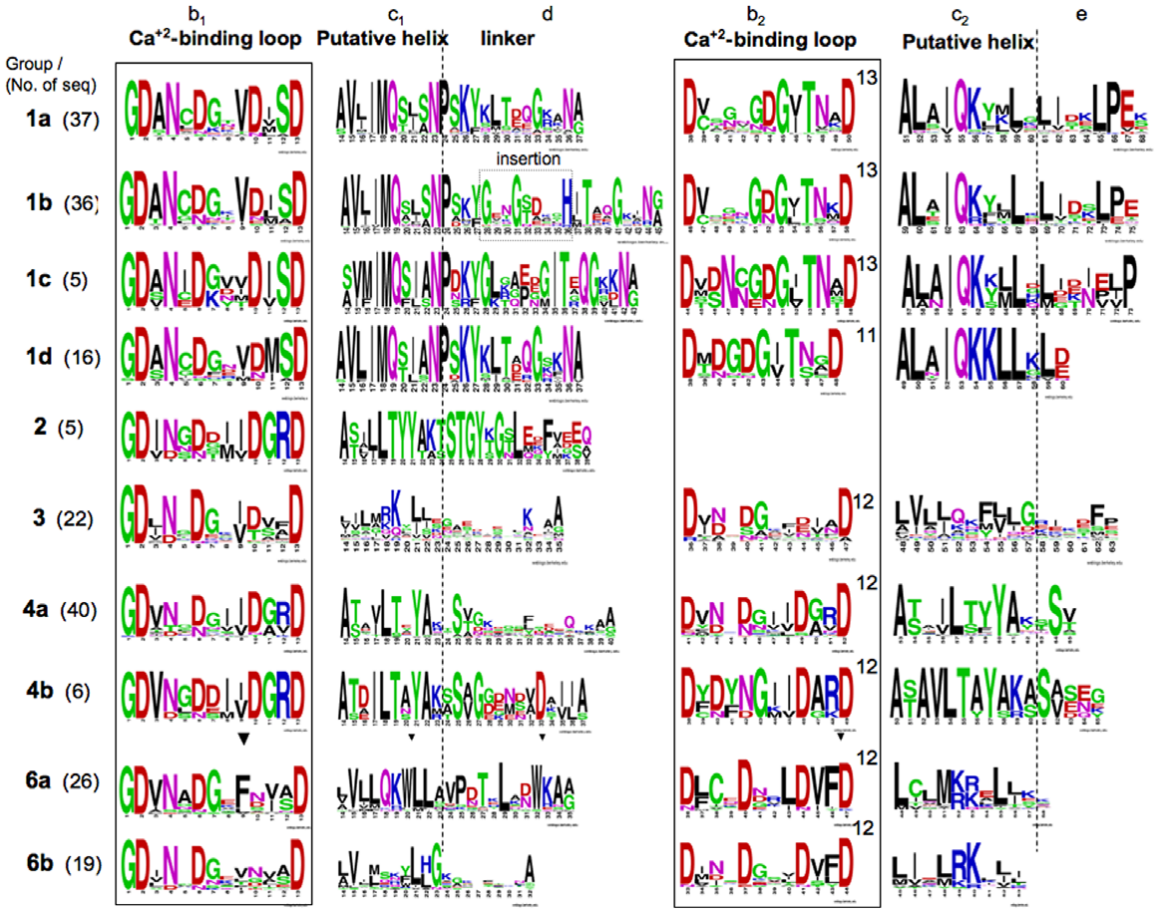
Conservation patterns of different dockerin groups from *R. flavefaciens* FD-1. The 222 dockerins were clustered into groups according to their conserved sequence features, and their sequence logo is presented. Segments along the dockerin modules (b–e at top) are labelled according to Pagés et al [32]. The length of the second repeat is marked for each group.

In order to identify dockerin-containing proteins in the *R. flavefaciens* FD-1 draft genome, we applied BLAST searches using homologous dockerins, and revealed 205 putative ORFs with complete dockerin modules, and 17 additional partial domains. The protein sequences of the dockerins are very diverse, and the sequence similarity between them ranges from 20 to 98%. However, most dockerin modules include all the characteristic segments as described earlier [32], including two Ca^2+^-binding repeats (segments b1 and b2) and their flanking regions (putative helices c1 and c2, and segments d and e) (Fig. 1). Notably, almost all dockerins begin with the canonically conserved Gly residue, with the exception of three dockerins (ORF00614, ORF01191 and ORF02267), which start with either Pro, Arg or Ser, respectively.

### Categorization of dockerin types

Examination of the sequence conservation along the dockerin modules allowed further classification of the dockerins into five major groups and four sub-groups (Fig. 1, and Table S1). Dockerins in each group share common patterns of residues with significant sequence similarity, together with unique and conserved sequence features which were manually refined. These include common residues of the Ca^2+^-binding repeats or in their flanking regions, which distinguish between the groups.

The largest number of dockerins (96) are clustered into **group 1** (Fig. 1), which is further divided into four subgroups (a–d), based on conserved sequence features which distinguish the dockerins in each group. Group 1 dockerins include the characteristic sequences VxIMQxxxNP in segments c1, and ALxIQKxxL in segments c2. They have exceptional features compared to all other dockerins: they contain the longest region linking between the Ca^2+^-binding repeats, (segment d, 37–45 aa long), where the sequences in group 1b are the longest because of an insertion sequence within it. Group 1 dockerins have an atypical number of conserved residues in the second Ca^2+^-binding repeat (segment b2), which is usually 12 residues long. However, in group 1a, 1b and 1c in this Ca^2+^-binding repeat is 13 residues long and in group 1d it is 11 residues long. A BLAST search with all group 1 dockerins against all known dockerins shows that they are exclusive to *R. flavefaciens*, because their only homologs are dockerins from *R. flavefaciens strain* 17. Representatives of this group of dockerins from both FD-1 and R17 strains were previously shown to bind to the cohesins present in ScaA, and to additional ScaA-type cohesins that are also present in ScaB in *R. flavefaciens FD-1* [18], [20]. Of special interest is the cohesin-carrying protein ScaC (ORF03113) [20], [26], which is mostly similar to group 1b dockerins, although it contains two insertions that are absent from the other group members.

**Group 2** dockerins include a small number of sequences, which are not similar to any other known dockerin from the non-redundant database. Only the first Ca^2+^-binding repeat was identified in these dockerins, and the dockerin itself was located at the C-terminus of each protein. Therefore, they may be either partial, and thus non-functional, or they can represent a new type of a single-binding mode of attachment to cohesins.

Unlike the group 2 sequences, **group 3** contains full-length dockerin modules, some of which are “clostridial” in nature, i.e., homologous to dockerins of *Clostridium cellulovorans, C. papyrosolvens* and *C. cellulolyticum* (gi numbers: ZP_04807887.1, ZP_05497793.1 and YP_002505573.1, respectively). Group 3 dockerins exhibit unusually high sequence variation in segments c1 and d. Interestingly, one of its dockerins is a CE3B homologue, known to bind the cohesin of the ScaC adaptor protein [20]. In future work, it will be interesting to determine whether other group 3 dockerins also exhibit specificity of binding to the ScaC cohesin.

**Figure 2:**
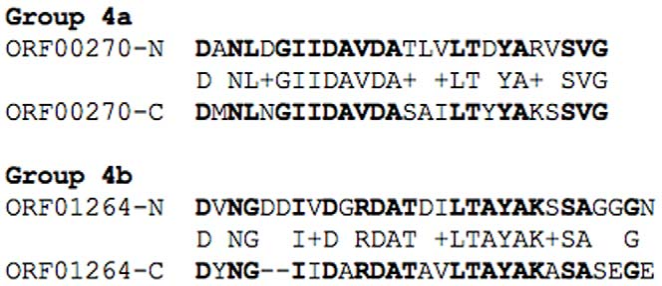
Internal symmetry within group 4 dockerins. The two putative calcium-binding repeats within each sequence were aligned for two representative dockerin sequences. Identical residues are shown in bold font.

**Group 4** dockerins are exclusive in *R. flavefaciens* FD-1, and do not have any known homologs in other bacteria. Comparison of the two repeating segments of the dockerins shows that group 4a and 4b dockerins are the only groups with internal sequence symmetry (Fig. 2). Dockerins of group 4a are similar to those from ScaB and CttA [28]. Newly recognised ScaB and CttA dockerins from *R. flavefaciens* 17 have been shown to bind to the cohesin of the cell-wall attached ScaE protein [20], [27], [28]. Group 4a dockerins are diverged from group 4b, which contain a shorter d segment, and also have a distinct conserved pattern in segments c1 and c2 (Fig. 1).

The six group 4b dockerins exhibit an interesting genomic organization. Five similar dockerins are arranged at the C-terminus of five ORFs (ORF01263-ORF01267), which are located head-to-tail in the same loci on the genome and may be regulated in a probable operon. These dockerin-containing proteins include a putative transglutaminase-like domain, and a signal peptide (Table 1). Interestingly, the C-terminal part of each protein is conserved among the five proteins (including regions upstream of the dockerin), while the N-terminal part is variable. Notably, their first Ca^2+^-binding repeat may be extended from 12 aa to 15 aa, due to the presence of a conserved Asp in position 16 of the dockerin. The additional group 4b dockerin (ORF01696) is similar to the other sequences, however, it is not in the same loci with them.

**Table 1:** Genes of dockerin-containing proteins in putative operons in *R. flavefaciens* FD-1.

Protein modules^a^	Dockerin group	Expression fold change^b^	ORF
SIGN-GH9-CBM3-Doc	1a	4.49	ORF01132
SIGN-CBM4-IgX-GH9-Doc	1a	6.60	ORF01133
SIGN-GH43-UNK-CBM13-Doc	1b	0.97	ORF00226
SIGN-CBM35-GH5-Doc	1b	2.11	ORF00227
SIGN-UNK-Doc	1b	1.17	ORF00228
SIGN-UNK-Doc	4a	0.60	ORF02170
SIGN-UNK-Doc	4a	0.66	ORF02171
SIGN-UNK-Doc	4a	1.07	ORF02172
SIGN-UNK-Doc	4a	0.98	ORF02173
SIGN-Transglutaminase like-Doc	4b	4.11	ORF01263
SIGN-Transglutaminase like-Doc	4b	3.86	ORF01264
SIGN-Transglutaminase like-Doc	4b	2.98	ORF01265
SIGN-Transglutaminase like-Doc	4b	3.52	ORF01266
SIGN-LRR-Doc	4b	3.87	ORF01267
SIGN-LRR-Doc	6a	2.09	ORF01964
SIGN-UNK(LbetaH-LamGL)-Doc	6a	1.80	ORF01965
SIGN-Doc-SERPIN	6b	1.16	ORF01368
SIGN-Doc-SERPIN	6b	N/A	ORF01369

a Abbreviations: sign, signal peptide; GH, glycoside hydrolase; CBM, carbohydrate-binding module; Doc, dockerin; unk, unknown; LRR, leucine-rich repeat.

b Expression data was based on Berg Miller *et al* [24].

**Figure 3:**
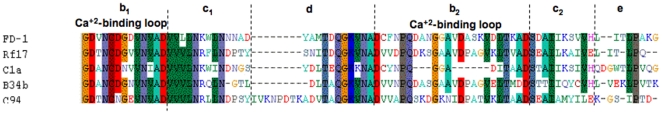
Conservation of ScaA proteins from different *R. flavefaciens* strains. Protein sequences were adapted from Jindou et al [25]. The N-terminal parts of the sequences are more conserved than the C-terminal part, and the second calcium-binding repeat could not be recognised. Thus, the labels of the dockerin modules (b–e at top) are approximated.

The dockerin of the ScaA protein is classified in its own group (**group 5**) as a single member and has unique sequence features, which differ from those of all other groups. The classification of a single dockerin in a single group is warranted in this case, owing to the central role that the parent protein plays in cellulosome architecture and the inclusion of its gene in the major scaffoldin gene cluster. Comparison of the ScaA dockerin among other *R. flavefaciens* strains (Fig. 3) shows a conserved N-terminus and a few variable positions at its C-terminus.

In the **group 6**a dockerins, conserved Phe (in segments b1 and b2) and Trp (in c1 and d) residues were detected. Not all of these residues are conserved in group 6b, which contains dockerins with the shortest d segment of all dockerins (32 residues). Dockerins from these groups are homologous to a few dockerins from Clostridium and Ruminococcus species.

After categorization of the *R. flavefaciens* dockerins into the above groups and sub-groups, only eight additional dockerins remained, which could not be attributed to any of the groups, owing to the very low sequence similarity.

### Gene expression levels according to different dockerin groups

**Figure 4:**
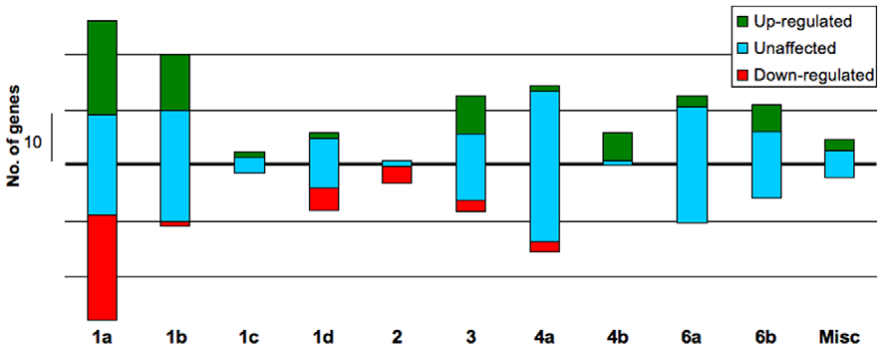
Gene expression levels of dockerin-containing genes. Number of up-regulated (green), down-regulated (red) or unaffected (blue) genes is marked for each group of dockerins. Expression data were determined for cultures grown on either cellulose [0.1% w/v pebble milled cellulose (filter paper)] or cellobiose (0.4% w/v) as a carbon and energy substrate, based on Berg Miller *et al* [24], where fold changes greater than or equal to 2-fold were considered up-regulated and fold changes less than or equal to 0.5-fold were considered down-regulated.

Inspection of the microarray data [24]) revealed the overall status of gene expression levels in the different dockerin groups (Fig. 4). As reported earlier, the expression levels of the majority (60%) of the dockerin-containing genes remained unaffected. However, as shown in the figure, most of the groups exhibit both up- and down-regulated genes. In some cases (i.e., groups 1c, 4b, 6a, 6b and the miscellaneous group of dockerins), no down-regulated genes were observed. In others, a bias towards up- (groups 1b and 3) or down-regulated genes (groups 1d and 2) was apparent. Interestingly, the genes in group 1a are almost equally distributed among the three categories (up, down and unaffected).

In several cases, genes of a single group appeared to be clustered into the same genomic locus (Table 1). This pattern was observed for certain genes in groups 1a, 1b, 4a, 4b, 6a and 6b, but not for those in groups 1c, 1d, 2 and 3. Some of these vicinal genes may be co-regulated, based on the microarray data [24], and these gene clusters may thus comprise operons.

### Characteristics of dockerin-containing proteins and their modules

Most of the ORFs of proteins containing complete dockerin modules have an N-terminal signal peptide. Among the predicted complete gene products, dockerins were typically located at the C-terminus (in 156 proteins), while in 32 other proteins the dockerins were internal, and in 19 cases were located at the N-terminus (following the signal peptide).

**Figure 5:**
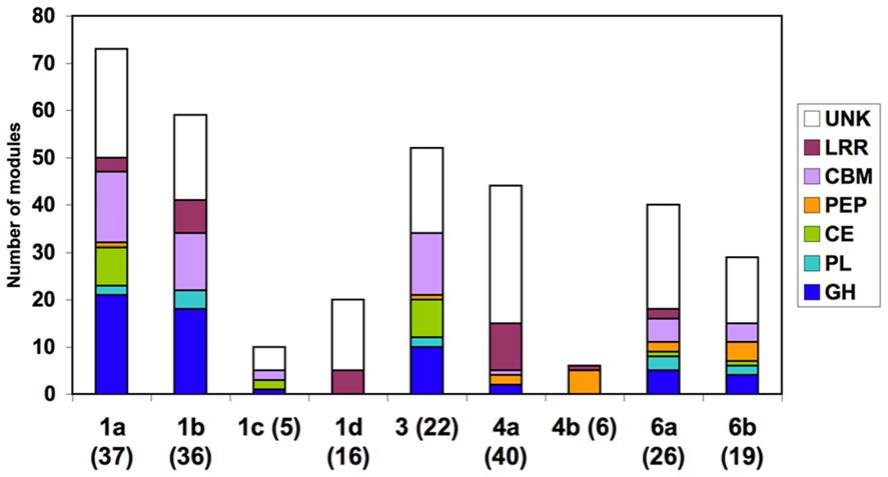
Catalytic and non-catalytic modules associated with different groups of dockerin in *R. flavefaciens* FD-1. Dockerin groups are shown on the x axis (number of encoded proteins carrying each type of dockerin in parentheses). UNK, unknown function; LRR, leucine rich repeats; CBM, carbohydrate binding modules; PEP, peptidases; CE, carbohydrate esterases; PL, pectate lyases; GH, glycoside hydrolases The small number of dockerins of group 2 and 5 not shown (but see Table 2).

The occurrence of both catalytic and non-catalytic (structural) modules in dockerin-containing proteins was analyzed further, within the context of the different dockerin groups (Table 2 and Fig. 5). In total, ∼50% of the dockerins were detected together with carbohydrate-active enzymes, including glycoside hydrolase modules, polysaccharide lyases and carbohydrate esterases. The total number of modules in the different categories of Table 2 exceeds the number of sequences (222) owing to the multi-modular nature of the dockerin-containing proteins. Unlike the other known genomes from cellulosome-producing bacteria, a significant number of dockerins were associated with protein modules annotated as putative peptidases. Moreover, numerous CBMs and cohesin-containing structural proteins (scaffoldins) were distributed among the dockerin-bearing proteins. Most of these modules appeared in dockerin-containing proteins, whose dockerins belong to groups 1a, 1b, 3 and 6. Notably, a great number of domains/modules of unknown function were observed. However, the distribution of different modules varied among the dockerin groups, as described below.

**Table 2:** Association of protein modules with different dockerins in *R. flavefaciens* FD-1.

Dockerin group:	*1a*	*1b*	*1c*	*1d*	*2*	*3*	*4a*	*4b*	*5*	*6a*	*6b*	*Misc.*	*Total*
**No. sequences:**	***37***	***36***	***5***	***18***	***5***	***21***	***40***	***6***	***1***	***26***	***19***	***8***	***222***
GH2		1											**1**
GH5	7	3 (4)^a^				1					1		**13**
GH9	4	3	1										**8**
GH10	3					2						1	**6**
GH11	4 (6)	1				1						1 (2)	**10**
GH16		1 (3)					1					1	**5**
GH24							1						**1**
GH26	2	1								1	1		**5**
GH43		2				3				1	1	1 (2)	**9**
GH44	1									1			**2**
GH48	1												**1**
GH53						1							**1**
GH74		1											**1**
GH97										1	1		**2**
GH98		1											**1**
***Total Hydrolases***	***24***	***17***	***1***	***0***	***0***	***8***	***2***	***0***	***0***	***4***	***4***	***6***	***66***
PL1	1	1								2	2		**6**
PL9	1												**1**
PL11		3				1				1			**5**
***Total Lyases***	***2***	***4***	***0***	***0***	***0***	***1***	***0***	***0***	***0***	***3***	***2***	***0***	***12***
CE1	1					3					1		**5**
CE3	2	2				3				1			**8**
CE4	1									1		1	**3**
CE12	2 (4)					1							**5**
CE15						1							**1**
***Total Esterases***	***8***	***2***	***0***	***0***	***0***	***8***	***0***	***0***	***0***	***2***	***1***	***1***	***22***
CBM3	3	1											**4**
CBM4	1	2	1										**4**
CBM6						3				1			**4**
CBM11	1												**1**
CBM13	3	5 (6)											**9**
CBM22	5 (6)					9 (11)							**17**
CBM32											2 (3)		**3**
CBM35	4	1				1				3 (4)	1		**11**
EndB	1									1			**2**
Undefined family	2	4	1				1						**8**
***Total CBM***	***21***	***14***	***2***	***0***	***0***	***15***	***1***	***0***	***0***	***6***	***4***	***0***	***63***
Transglutaminase	1					1	1	5			2		**10**
Peptidase							2				1		**3**
Metalloprotease										1			**1**
Carboxypeptidase										1			**1**
VanY protease											1		**1**
***Total Peptidases***	***1***	***0***	***0***	***0***	***0***	***1***	***3***	***5***	***0***	***2***	***4***	***0***	***16***
Cohesin		2		1 (2)			4 (12)		1				**17**
Leucine-rich Rpt	3	6		5	1		10	1		2			**28**
SERPIN		1									2		**3**
Unknown	20(23)	13(18)	4(5)	10(15)	3(5)	13(18)	24(29)	0	0	17(22)	10(14)	4(6)	**155**

a Total numbers of modules given in parenthesis include those occurring more than once in the same protein.

In terms of the content of carbohydrate-active enzymes [33], groups 1a, 1b and 3 were significantly enriched in the numbers of glycoside hydrolases, carbohydrate esterases and associated CBMs. Notably, most if not all of the genuine dockerin-containing cellulases (i.e., confirmed endo- and exoglucanases) were members of group 1a and 1b, and most of these were associated with group 1a. Group 1a dockerin-containing enzymes included the sole critical GH48 exoglucanase, four GH9 endoglucanases and six GH5 cellulases (an additional GH5 enzyme is annotated as a xylanase). Group 1a also contained most of the family 10 and 11 xylanases, two GH26 mannanases and a GH44 enzyme (related to the well-documented EndB enzyme from *R. flavefaciens* strain 17 [17]). Group 1b contained the remainder of the cellulases; two from family 5 and three from family 9. The other enzymes that bear group 1b dockerins were annotated as hemicellulases, including the lone GH74 xyloglucanase. In contrast, all of the parent enzymes associated with the group 3 dockerins were putative hemicellulases. Similarly, most or all of the group 6a and 6b dockerin-containing enzymes were annotated as hemicellulases from various families.

Most (14 out of 20) of the proteins carrying carbohydrate esterase (CE) modules were classified as multifunctional proteins (Table 3), which will be discussed in the subsequent section. Conversely, most (8 of 11) of the dockerin-bearing polysaccharide lyases (PLs) did not carry an additional catalytic module. In both cases, their distribution into the dockerin groups was rather similar to that of the GH-bearing enzymes. None of the dockerins in this category belonged to groups 1c, 1d, 2, 4a or 4b.

**Table 3:** Association of multi-functional proteins with different dockerin groups in *R. flavefaciens* FD-1.

Protein modules^a^	Dockerin group	Expression fold change^b^	ORF
SIGN-GH11-CBM22-GH10-Doc-GH11	1a	4.74	ORF00468
SIGN-UNK-PL1-UNK-PL9-UNK(CBM)-Doc	1a	1.88	ORF00696
SIGN-GH11-CBM13-CE1-Doc	1a	9.79	ORF00775
SIGN-GH11-CBM22-GH10-Doc-CBM22-CE4	1a	63.01	ORF01222
SIGN-GH11-CBM22-Doc-GH11-CE3	1a	25.32	ORF01315
SIGN-UNK-CE12-CBM13-Doc-CBM35-CE12	1a	4.01	ORF02983
SIGN-UNK-CE12-CBM13-Doc-CBM35-CE12	1a	7.32	ORF03219
SIGN-Doc-GH16-GH16-GH16	1b	2.21	ORF00265
SIGN-GH5-CBM(EndA)-GH5-Doc	1b	4.97	ORF01388
SIGN-CE8-UNK-PL1-UNK-Doc	1b	1.44	ORF02371
SIGN-GH43-X19-CBM22-Doc-CE1	3	2.42	ORF00341
SIGN-GH43-CBM6-CBM22-Doc-CE1	3	4.12	ORF00764
SIGN-GH53-CE3-Doc	3	1.07	ORF01739
SIGN-CE3-CBM22-Doc-CE15	3	8.56	ORF02390
SIGN-PL11-Doc-CBM35-CE12	3	1.66	ORF03451
UNK-CBM22-GH10-CBM22-Doc-GH43-CBM6	3	8.81	ORF03865
SIGN-CBM35-Esterase/Lipase-Doc-CBM35-GH26	6a	0.74	ORF03447
SIGN-GH11-CBM22-GH10-Doc-GH11-CE4	Unclassified	50.1	ORF03896

a Abbreviations: sign, signal peptide; GH, glycoside hydrolase; CBM, carbohydrate-binding module; Doc, dockerin; unk, unknown; PL, polysaccharide lyases; CE, carbohydrate esterases; LRR, leucine-rich repeat.

b Expression data was based on Berg Miller *et al* [24].

One of the intriguing features apparent in the *R. flavefaciens* cellulosome is the relatively high number of putative proteases in dockerin-containing proteins, which contrasts sharply with the genome of *C. thermocellum*. Interestingly, about half of these appended dockerins belong to group 4. Significantly, 5 of the 6 dockerins from group 4b are attached to the proteins annotated as transglutaminase-like enzymes as described above.

Even more intriguing is the number of dockerin-containing proteins that contain regions lacking similarity to known proteins and thus designated “unknown”. This includes 118 different proteins representing 155 putative domains of unknown function. Again, the presence of such a large number of unknown domains in putative cellulosomal proteins of *R. flavefaciens* is in stark contrast to the genome of *C. thermocellum*, in which only eight unknown dockerin-containing proteins are evident.

### Multi-functional architecture of dockerin-containing proteins

Dockerin-containing proteins that contained more than one catalytic module (i.e., GH, PL and/or CE) were observed mainly in groups 1a, 1b and 3, and were particularly apparent among dockerin-carrying xylanases and other hemicellulases (Table 3). Most of these proteins were up-regulated in cells grown on microcrystalline cellulose (versus cellobiose), sometimes to excess, and none were down-regulated. These observations underscore the significance of this complex set of multi-functional enzymes and their importance in the degradation of recalcitrant cellulosic plant cell wall polysaccharides, in this fiber-degrading rumen bacterium.

### Proteins carrying cohesins and cohesin-like modules

By definition, cellulosomal cohesin-containing proteins are classified as scaffoldins that play a structural role in cellulosome architecture. Not all of the putative cohesin-containing scaffoldins contain dockerins, and several suspected *R. flavefaciens* cohesin modules remain unconfirmed. Only two of these scaffoldins, ScaC and a ScaE-like protein, contain dockerins that are members of a group (1b), which would presumably bind large numbers of carbohydrate-active enzymes. The others are distributed in groups, in which such enzymes are either rare or lacking altogether. Four confirmed cohesin-carrying proteins that are encoded by the *sca* gene cluster were described in previous publications [22] [25]. The general organization of the gene cluster in the different *R. flavefaciens* strains is identical, although the sequences and modular structure of the proteins differ. The scaffoldin proteins encoded by the *sca* gene cluster constitute the backbone of cellulosome architecture in this bacterium. Three of them, ScaA, ScaB and ScaC, carry C-terminal dockerins, while ScaE carries a cell surface attachment signal motif, but no dockerin. In contrast to ScaC, the dockerins of ScaA and ScaB belong to groups 5 and 4, respectively.

## Discussion

Genomic analysis of *R. flavefaciens* FD-1 has revealed the most diversified and elaborate cellulosome complex thus far discovered. The cellulosome of this bacterial strain possesses an unprecedented number of dockerin-containing proteins (at least 222), when compared with other cellulolytic bacteria. The genome of *C. thermocellum,* for example, contains only about 76 dockerin-carrying cellulosome components [22], and that of the mesophilic strain *C. cellulolyticum* contains 71 putative dockerins (unpublished results). Unlike the dockerins of both the *C. thermocellum* and *C. cellulolyticum* cellulosomes, where the great majority are very similar in their sequences, the dockerin sequences of the *R. flavefaciens* FD-1 cellulosome can be divided into distinctive groups based on sequence divergence.

Due to the wealth of the latter sequences, the various sequence features have been approached here by both bioinformatics tools and manual inspection, in order to provide further insight into their interrelationship and possible function. Some of the groups clearly have distinct sequence patterns, which are conserved within a few dozen dockerin modules, even though the latter originate from different parent proteins. The distribution of catalytic modules, binding modules and dockerin sequences predicted from the *R. flavefaciens* FD-1 draft genome confirms a dominant role for cellulosome organization among extracellular enzymes that are concerned with plant cell wall breakdown by this bacterium.

The general role that is recognised for dockerins and cohesins is in mediating specific protein-protein interactions that result in the assembly of the multienzyme cellulosome complex. In another strain of this species, *R. flavefaciens* 17, previous studies have shown direct functional evidence for four specific interactions: (i) EndB-type dockerins with ScaA-type cohesins; (ii) the ScaB dockerin with the ScaE cohesin; (iii) the ScaA dockerin with ScaB-type cohesins; and (iv) the CE3B dockerin with the ScaC cohesin (Jindou et al 2006). However, these interactions likely represent but a portion of the total set of interactions, since the genome of strain 17 has yet to be sequenced; our current knowledge of the different types of cohesin-dockerin pairs is thus confined to the relatively small number of cellulosomal components thus far sequenced and the paucity of relevant experimental evidence thus far accumulated. Genome sequencing of *R. flavefaciens* FD1 has served both to broaden greatly the number of sequences available for this species and to emphasise that the known homologous sequences between the two strains are decidedly different. Homologs of some of the *R. flavefaciens* 17 dockerins were found to belong to distinct dockerin groupings characterised for *R. flavefaciens* FD-1. Thus, the EndB dockerin of strain 17 belongs to group 1, the ScaB dockerin to group 4a, the CE3B dockerin to group 3 and the ScaA dockerin to group 5.

The uniquely large number of *R. flavefaciens* FD-1 dockerins and their patterns of conservation may reflect a mechanistic diversity of different cohesin-dockerin interactions. Truncated dockerins (such as these classified in group 2) may be active via a single-binding mode, while other types of dockerins may be recruited to specific polysaccharide-degrading functions of the bacterium, which are exclusive to the ruminal environment and were therefore not developed in other cellulolytic bacteria. It is also logical to assume that many dockerins were presumably subjected through evolution to strong selection forces and were duplicated within each group (for example in group 4b), thereby expanding the repertoire of dockerins in *R. flavefaciens*. Different cohesin-dockerin pairings may then reflect different evolutionary origins, with adaptive changes in *R. flavefaciens* helping to organise these different components into enzyme complexes that function effectively on a wide variety of plant cell wall material under the changing conditions of the rumen environment. Moreover, the different cohesin-dockerin pairings could play a critical role in structuring the complexes and in regulating the inclusion of the parent protein (notably the enzymes) into the complex in response to environmental signals. The numerical ratio in the same bacterium of a few dozens cohesins versus the two hundred dockerins reflects the key modular nature of cellulosomal structures and their complexity. Finally, it is entirely possible that certain interactions, for example some of those involving the group 4 dockerins, might play roles that are not directly related to cellulosome function, but to other unknown functions, perhaps including the structuring of the bacterial cell surface [34].

The patterns which were observed in the dockerin-containing proteins provide another level of complexity to the *R. flavefaciens* FD-1 cellulosome. On the one hand, a large number of unknown domains were detected among all groups of dockerins as opposed to the status of the *C. thermocellum* genome, but on the other, catalytic modules (glycoside hydrolases, polysaccharide lyases, carbohydrate esterases and associated CBMs) were particularly associated with only a few select groups (1a, 1b and 3). Attempts to understand this complexity included inspection of the levels of gene expression, which mainly revealed that multi-modular proteins were mostly up-regulated in cells grown on cellulose versus growth on cellobiose.

It is not possible, however, to simply equate dockerin clusters with their specificities. This question can only be answered through careful and extensive functional studies on the interactions between purified modules, and on the determinants of binding specificity. The current study provides the rationale for such experiments. The conserved differences between the different dockerins may be eventually reflected in their stability, function or expression within the context of the parent protein, in response to their role in the rumen environment.

## Supporting Information

Table S1 Assignment of dockerin ORFs to their groups.
(0.14 MB DOC)
