# Supplementary material for: Abundance and Diversity of Dockerin-Containing Proteins in the Fiber-Degrading Rumen Bacterium, Ruminococcus flavefaciens FD-1
Source: PLoS One. 2010 Aug 30;5(8):e12476. doi: 10.1371/journal.pone.0012476 (PMC2930009; doi:10.1371/journal.pone.0012476)
Supplement: Table S1 — Assignment of dockerin ORFs to their groups. (0.14 MB DOC) [file pone.0012476.s001.doc]

**Table S1: Assignment of dockerin ORFs to their groups.**

| **1a** | **1b** | | **1c** | | **1d** | | **2** | | **3** | **4a** | **4b** |
| --- | --- | --- | --- | --- | --- | --- | --- | --- | --- | --- | --- |
| ORF00059 | ORF00082 | | ORF00463 | | ORF00782 | | ORF00690 | | ORF00341 | ORF00078 | ORF01696 |
| ORF00389 | ORF00226 | | ORF00535 | | ORF00810 | | ORF01750 | | ORF00381 | ORF00081 | ORF01263 |
| ORF00412 | ORF00227 | | ORF02440 | | ORF01217 | | ORF02067 | | ORF00408 | ORF00251 | ORF01264 |
| ORF00468 | ORF00228 | | ORF03330 | | ORF02142 | | ORF03182 | | ORF00464 | ORF00270 | ORF01265 |
| ORF00696 | ORF00265 | | ORF03471 | | ORF02187 | | ORF03195 | | ORF00691 | ORF00384 | ORF01266 |
| ORF00762 | ORF00414 | |  | | ORF02267 | |  | | ORF00764 | ORF00409 | ORF01267 |
| ORF00775 | ORF00471 | |  | | ORF02556 | |  | | ORF01323 | ORF00438 |  |
| ORF01053 | ORF00645 | |  | | ORF03067 | |  | | ORF01425 | ORF00534 |  |
| ORF01082 | ORF00767 | |  | | ORF03070 | |  | | ORF01739 | ORF00614 |  |
| ORF01083 | ORF01045 | |  | | ORF03084 | |  | | ORF01912 | ORF00658 |  |
| ORF01132 | ORF01050 | |  | | ORF04060 | |  | | ORF01934 | ORF00700 |  |
| ORF01133 | ORF01321 | |  | | ORF04065 | |  | | ORF02174 | ORF00794 (ScaH) |  |
| ORF01222 | ORF01327 | |  | | ORF04066 | |  | | ORF02390 | ORF01105 |  |
| ORF01315 | ORF01328 | |  | | ORF04069 (ScaJ) | |  | | ORF03451 | ORF01341 |  |
| ORF01326 | ORF01384 | |  | | ORF04079 | |  | | ORF03454 | ORF01672 |  |
| ORF01857 | ORF01388 | |  | | ORF04083 | |  | | ORF03729 | ORF01674 |  |
| ORF01869 | ORF01453 (similar to ScaE coh) | |  | | ORF04108 | |  | | ORF03865 | ORF01863 |  |
| ORF01926 | ORF01753 | |  | | ORF04111 | |  | | ORF04012 | ORF02050 |  |
| ORF02064 | ORF01867 | |  | |  | |  | | ORF04092 | ORF02062 |  |
| ORF02186 | ORF01872 | |  | |  | |  | | ORF04112 | ORF02065 |  |
| ORF02444 | ORF02371 | |  | |  | |  | | ORF04165 | ORF02170 |  |
| ORF02518 | ORF02389 | |  | |  | |  | |  | ORF02171 |  |
| ORF02519 | ORF02868 | |  | |  | |  | |  | ORF02172 |  |
| ORF02975 | ORF02877 | |  | |  | |  | |  | ORF02173 |  |
| ORF02981 | ORF02883 | |  | |  | |  | |  | ORF02202 |  |
| ORF02983 | ORF02890 | |  | |  | |  | |  | ORF02449 |  |
| ORF03006 | ORF02978 | |  | |  | |  | |  | ORF02869 |  |
| ORF03219 | ORF03018 | |  | |  | |  | |  | ORF02894 |  |
| ORF03338 | ORF03113 (ScaC) | |  | |  | |  | |  | ORF03115 (ScaB) |  |
| ORF03455 | ORF03228 | |  | |  | |  | |  | ORF03116 (cttA) |  |
| ORF03910 | ORF03414 | |  | |  | |  | |  | ORF03129 (ScaF) |  |
| ORF03925 | ORF03577 | |  | |  | |  | |  | ORF03132 |  |
| ORF03970 | ORF03829 | |  | |  | |  | |  | ORF03218 |  |
| ORF03975 | ORF03976 | |  | |  | |  | |  | ORF03491 |  |
| ORF03977 | ORF04149 | |  | |  | |  | |  | ORF03737 |  |
| ORF03978 | ORF04293 | |  | |  | |  | |  | ORF03749 |  |
| ORF04344 |  | |  | |  | |  | |  | ORF03750 |  |
|  |  | |  | |  | |  | |  | ORF04062 |  |
|  |  | |  | |  | |  | |  | ORF04333 (ScaI) |  |
|  |  | |  | |  | |  | |  | ORF04343 |  |
| **5** | | **6b** | | **6a** | | **Unclassified** | |  | | | |
| ORF03114 (ScaA) | | ORF00232 | | ORF00886 | | ORF00046 | |  | | | |
|  | | ORF00444 | | ORF00903 | | ORF00769 | |  | | | |
|  | | ORF00659 | | ORF01307 | | ORF01117 | |  | | | |
|  | | ORF00728 | | ORF01309 | | ORF01191 | |  | | | |
|  | | ORF00942 | | ORF01312 | | ORF01442 | |  | | | |
|  | | ORF01368 | | ORF01475 | | ORF02270 | |  | | | |
|  | | ORF01369 | | ORF01478 | | ORF02575 | |  | | | |
|  | | ORF01486 | | ORF01568 | | ORF03896 | |  | | | |
|  | | ORF01513 | | ORF01570 | |  | |  | | | |
|  | | ORF01541 | | ORF01650 | |  | |  | | | |
|  | | ORF01804 | | ORF01654 | |  | |  | | | |
|  | | ORF01913 | | ORF01964 | |  | |  | | | |
|  | | ORF02549 | | ORF01965 | |  | |  | | | |
|  | | ORF02617 | | ORF02224 | |  | |  | | | |
|  | | ORF02712 | | ORF02889 | |  | |  | | | |
|  | | ORF03223 | | ORF02965 | |  | |  | | | |
|  | | ORF03229 | | ORF03141 | |  | |  | | | |
|  | | ORF03340 | | ORF03305 | |  | |  | | | |
|  | | ORF03897 | | ORF03307 | |  | |  | | | |
|  | |  | | ORF03447 | |  | |  | | | |
|  | |  | | ORF03449 | |  | |  | | | |
|  | |  | | ORF03462 | |  | |  | | | |
|  | |  | | ORF03573 | |  | |  | | | |
|  | |  | | ORF03579 | |  | |  | | | |
|  | |  | | ORF03670 | |  | |  | | | |
|  | |  | | ORF03682 | |  | |  | | | |
